# The Effect of Accessory Pathway Location on Cardiac Function in Adult Patients with Wolff–Parkinson–White Syndrome

**DOI:** 10.1155/2021/8841736

**Published:** 2021-01-05

**Authors:** Ying Zhang, Mei Xin, Tongbao Liu, Shangming Song, Wenxin Wang, Jun Li, Bo Xu, Xiaoyang Hou, Bo Dong

**Affiliations:** ^1^Department of Cardiology, Shandong Provincial Hospital Affiliated to Shandong First Medical University, Jinan 250012, Shandong, China; ^2^Department of Cardiology, Shandong Provincial Hospital Affiliated to Shandong University, Jinan 250012, Shandong, China; ^3^Department of Cardiology, The Fifth People's Hospital of Jinan, Jinan 250022, Shandong, China; ^4^Department of Intensive Care Unit, Qinghai Red Cross Hospital, Xining 810000, Qinghai, China

## Abstract

**Introduction:**

The relationship between ventricular pre-excitation and left ventricular dysfunction has been described in the absence of sustained supraventricular tachycardia in a series of case reports. However, there have been no systematic studies about the effect of ventricular pre-excitation on cardiac function in adult patients with different accessory pathway locations.

**Methods and Results:**

Patients were divided into four groups based on the type and location of their accessory pathway: septal, right free wall, left free wall, and concealed. N-terminal pro-B-type natriuretic peptide (NT-proBNP) levels, electrocardiogram recordings, electrophysiological properties, and transthoracic echocardiographic data (septal-to-posterior wall motion delay (SPWMD) and interventricular mechanical delay (IVMD) indicating intraventricular and interventricular dyssynchrony) were compared before and after successful ablation. Before radiofrequency catheter ablation, left ventricular ejection fraction (LVEF) was significantly lower in patients with septal and right free wall accessory pathways. Within three months after radiofrequency catheter ablation, NT-proBNP levels decreased, left ventricular function improved, and intraventricular left ventricular dyssynchrony disappeared. There was a negative correlation between initial LVEF with initial QRS duration and initial SPWMD. Notably, SPWMD had a stronger correlation with LVEF than initial QRS duration.

**Conclusions:**

Anterograde conduction with a septal or right free wall accessory pathway may cause left ventricular dyssynchrony and impair left ventricular function. Intraventricular left ventricular dyssynchrony seems to be responsible for the pathogenesis of left ventricular dysfunction. Radiofrequency catheter ablation results in decreased NT-proBNP levels, normalized QRS duration, mechanical resynchronization, and improved left ventricular function.

## 1. Introduction

Wolff–Parkinson–White (WPW) is a cardiac pre-excitation syndrome caused by an accessory pathway (AP) that bypasses the atrioventricular node and is associated with supraventricular tachycardia [1]. Incessant or recurrent AP-mediated supraventricular tachycardia (SVT) can cause dilated cardiomyopathy [2, 3]. Nevertheless, it has been reported that some patients with overt ventricular pre-excitation, either children or adults, develop left ventricular dysfunction and dilated cardiomyopathy in the absence of documented SVT [4–6]. Pre-excitation through the AP might affect left ventricular wall motion, and thus the extent and location of the pre-excited myocardium could be important determinants of global ventricular function. If true, suppression or elimination of pre-excitation can reverse the remodeling of ventricular mechanics. However, there have been no systematic studies on the effect of ventricular pre-excitation with different accessory pathway locations on cardiac function, especially in adult patients exposed to the accessory pathway for an extended period.

Radiofrequency catheter ablation (RFA) of AP is an established treatment for symptomatic WPW syndrome, but its application in asymptomatic patients remains controversial [7]. Also, a recent study showed that left ventricular (LV) dyssynchrony in asymptomatic WPW syndrome is a risk factor for developing dilated cardiomyopathy [8]. However, the utility of RFA for reducing or eliminating pre-excitation-induced dyssynchrony is unclear.

The present study aimed to determine the effect of accessory pathway location on cardiac function in adult patients and assess the extent of reverse remodeling produced by RFA. Additionally, we examined the relationship between cardiac functions with cardiac dyssynchrony and initial QRS duration.

## 2. Methods

### 2.1. Study Patients and Design

A total of 80 consecutive patients with WPW syndrome who received catheter ablation in our hospital from January 2018 to June 2019 were included.

Inclusion criteria were as follows. Patients with diagnostic criteria for WPW [9], aged from 18 to 70 years old, were enrolled. All patients had normal sinus rhythm and no tachycardia for at least one week. None had a cumulative arrhythmia burden of five hours per month. All patients stopped antiarrhythmic drugs for a period of at least five half-lives of these drugs before ablation.

Exclusion criteria were as follows. We excluded patients with known cardiac or noncardiac disease, including hypertension, diabetes, hyperthyroidism, liver or kidney dysfunction, or acute infection. Patients with bundle branch block or patients with multiple accessory pathways and intermittent pre-excitation assessed during electrophysiological studies were also excluded.

Patients were divided into four groups based on the location of the AP: septal, right free wall, left free wall, and concealed. During recruitment, once 20 patients are enrolled in any group, the enrollment stops in that group. The study met medical ethical standards and has been approved by the ethics committee. Written informed consent was obtained from each patient.

Demographic data were obtained from the medical records. Laboratory data, including NT-proBNP levels, were assayed using standard laboratory procedures at the Department of Clinical Laboratory of our hospital.

### 2.2. Electrocardiogram

Twelve-lead ECG during sinus rhythm was obtained in all patients before and after RFA with a Mac 5000 System (GE Medical Systems, Milwaukee, WI, USA). QRS duration measurements were automatically performed.

### 2.3. Echocardiography

Before catheter ablation and three months later, transthoracic echocardiography was performed by a single physician blinded to the ECG and electrophysiological findings. The end-diastolic LV diameter (LVEDd) was measured from parasternal long-axis M-mode recording. Left ventricular ejection fraction (LVEF) was calculated using the biplane Simpson's method. Interventricular mechanical delay (IVMD) was used to evaluate the mechanical delay between the left and right ventricles [10]. Septal-to-posterior wall motion delay (SPWMD), the difference in timing of the peak systolic motion between the anteroseptal wall and left posterior wall, was measured to assess intraventricular dyssynchrony [11].

### 2.4. Electrophysiological Study and RFA

Electrophysiological studies and RFA were carried out according to standard techniques [12]. Briefly, three quadripolar electrode catheters were inserted percutaneously and introduced to the high right atrium, His bundle position, and right ventricular apex via the femoral vein. A decapolar catheter was pushed into the coronary sinus. The following parameters were examined: AH interval during sinus rhythm, HV interval during sinus rhythm, tachycardia cycle length, anterograde effective refractory period of the accessory pathway, and retrograde effective refractory period of the accessory pathway. Standard electrophysiological actions determined the location of the accessory pathway. An 8Fr deflectable catheter with a 4 mm ablation tip (Biosense Webster, Diamond Bar, CA, USA) was inserted through the right femoral vein for mapping and catheter ablation. The radiofrequency current was set at a maximal power output of 30 W and an upper temperature limit of 55°C. The ablations of right-sided accessory pathways were accomplished using Swartz sheaths to stabilize the catheters. Radiofrequency ablation was considered successful, if the AP conduction disappeared at least 30 minutes after radiofrequency ablation. The long-term success of RFA was defined as the absence of tachycardia recurrence or pre-excitation patterns on the surface ECG three months after the procedure.

### 2.5. Statistical Analysis

Continuous variables are presented as mean ± standard deviation (SD). Differences between two groups were compared using Student's *t*-test while comparisons among more than two groups were conducted using one-way analysis of variance with Bonferroni's post hoc test. Categorical variables are presented in frequency (number) and proportion (%). The *χ*^2^ test was used to compare differences between groups. Correlations between nonparametric variables were tested using Pearson's correlation coefficient. A *P* value <0.05 was considered statistically significant. All statistical analyses were performed using SPSS 23.0 (SPSS, Chicago, IL, USA).

## 3. Results

### 3.1. General Clinical Characteristics

The clinical characteristics of the study population are summarized in Table 1. There were no significant differences among the four groups in terms of age, sex, weight, heart rate, and laboratory data, including blood urea nitrogen, creatinine, estimated glomerular filtration rate, high-sensitivity C-reactive protein, and cystatin C levels. None of the patients suffered from incessant tachycardia, rapid conduction of atrial fibrillation via the accessory pathways, or other arrhythmias that might have contributed to the reduced LV function.

### 3.2. Electrocardiogram Data

The surface 12-lead ECG after RFA showed the normalization of QRS duration and disappearance of *δ* wave (Table 2). In addition, all patients achieved a long-term success of RFA with no tachycardia recurrence during follow-up.

### 3.3. Echocardiographic Findings

**Figure 1:**
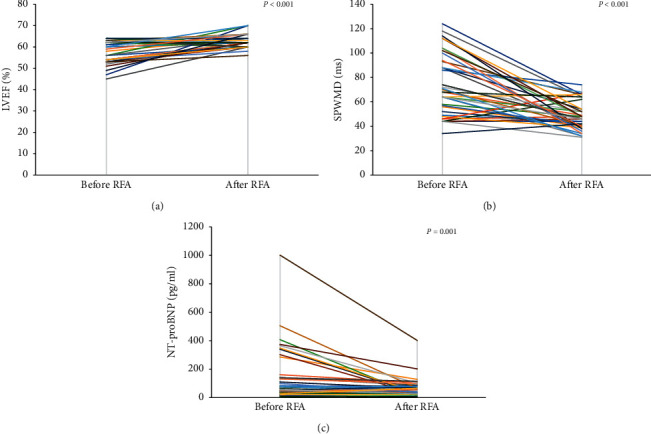
Pre- and postradiofrequency ablation findings for LVEF, SPWMD, and NT-proBNP in patients with septal and right free wall accessory pathways (*n* = 40). Radiofrequency ablation of accessory pathways resulted in increased LVEF (a), recovery of SPWMD (b), and decreased NT-proBNP levels (c).

Systolic functions among the four groups before and after ablation are shown in Table 2. There were significant differences in LVEF between the four groups (*P* < 0.001). LVEF was significantly lower in patients with septal accessory pathways and right free wall accessory pathways than in patients with left free wall accessory pathways and concealed accessory pathway, respectively (*P* < 0.001). Two patients (10% of the septal group) from the septal group and one patient (5% of the right free wall group) from the right free wall group had LVEF < 50%. None of the patients in the left free wall accessory pathway and concealed accessory pathway groups had LVEF < 50%. After ablation, LVEF increased significantly in septal and right free wall accessory pathway groups (*P* < 0.001; Figure 1(a)). There were no significant differences in LVEDd among the four groups before and after ablation.

The parameters reflecting LV synchrony in the four groups before ablation are shown in Table 2. Before ablation, intraventricular LV synchrony decreased in the septal and right free wall accessory pathway groups, which was demonstrated as a significant increase in SPWMD (*P* < 0.001). After ablation, SPWMD was significantly shorter in the septal and right free wall accessory pathway groups (*P* < 0.001, resp.; Figure 1(b)). No changes were observed after ablation in the other two groups.

Interventricular synchrony did not differ between the four groups before and after ablation, assessed by IVMD (*P*=0.092 and *P*=0.06, resp.).

### 3.4. Electrophysiological Data

During sinus rhythm, the AH intervals were not different among the four groups (Table 3). On the contrary, HV intervals were shorter in WPW patients compared with the concealed accessory pathway group. The effective refractory period of the accessory pathway and tachycardia cycle length was not different among the four groups. All RFA procedures were performed without complications.

### 3.5. Plasma NT-proBNP Levels in Patients

The levels of plasma NT-proBNP were significantly different among the four groups (*P*=0.039), with a higher NT-proBNP plasma concentration in the septal and right free wall accessory pathway groups compared with the concealed accessory pathway group (Table 1). After ablation, plasma NT-proBNP levels significantly decreased in the septal and right free wall accessory pathway groups (*P*=0.001; Figure 1(c)).

### 3.6. Correlations

**Figure 2:**
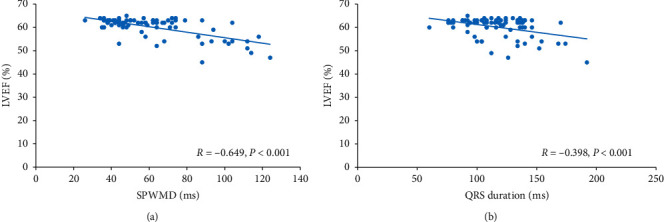
Correlation between LVEF and septal-to-posterior wall motion delay (SPWMD) or QRS duration in all patients (*n* = 80). There were negative correlations between initial LVEF and initial SPWMD (a) or initial QRS duration (b). Moreover, SPWMD had a higher correlation with LVEF than initial QRS duration.

There was a negative correlation between initial LVEF with initial SPWMD (Figure 2(a)) and initial QRS duration (Figure 2(b)). Notably, SPWMD had a stronger correlation with LVEF than initial QRS duration.

## 4. Discussion

The relationship between ventricular pre-excitation and left ventricular dysfunction has been described in the absence of sustained supraventricular tachycardia in a series of case reports [13–15]. This study report results from a group of adult patients with ventricular pre-excitation. The main findings are as follows: (1) patients with ventricular pre-excitation are at risk of developing LV dysfunction, even in the absence of long-standing and recurrent tachyarrhythmia; (2) LV function is influenced by AP location, being significantly lower in WPW patients with septal and right free wall accessory pathways; (3) intraventricular LV dyssynchrony appeared to be responsible for the pathogenesis of LV dysfunction; (4) radiofrequency ablation of accessory pathways resulted in normalization of QRS duration, decrease in NT-proBNP levels and mechanical resynchronization, and improved LV function within three months suggesting a causal relation between ventricular pre-excitation and development of LV dysfunction.

Relationship between the location of accessory pathway and left ventricular wall motion has been studied previously. It has been reported that septal accessory pathways result in the greatest degree of LV mechanical dyssynchrony, possibly because accessory pathways closer to the sinus node alter normal ventricular depolarization to a greater extent [11, 16]. Kwon et al. [11] observed the echocardiographic parameters of 62 patients with WPW syndrome before and after radiofrequency ablation to determine the extent to which the accessory pathway contributes to global LV dysfunction. They found that LVEF was significantly lower in the right-sided septal subgroup than the right- and left-sided free wall subgroups. Tomaske et al. [16] retrospectively compared LV function of 34 patients with right septal or posteroseptal accessory pathways before and after ablation. Before ablation, LVEF was impaired in 19 of their 34 patients. However, LVEF improved, and SPWMD decreased afterwards, indicating that WPW syndrome with right septal or posteroseptal accessory pathways may cause LV dyssynchrony and jeopardize global LV function. Chen et al. [17] used single-photon emission computed tomography (SPECT) to identify LV dyssynchrony in 22 of a cohort of 42 patients with ventricular pre-excitation; most of those with dyssynchrony had accessory pathways in the right-sided septal wall. Hishida et al. [18] reported that right and left free wall accessory pathways cause abnormal motion of the septal wall and left posterior wall, respectively. Park et al. reported that left free wall [19] accessory pathway caused severe intraventricular dyssynchrony. However, the accessory pathway locations that may cause cardiac dysfunction are still controversial.

Our results suggest that right-sided accessory pathways, especially septal accessory pathways, may impair LV function, manifested as elevated BNP levels and decreased LVEF. Also, there was a negative correlation between initial LVEF with initial QRS duration and initial SPWMD. Moreover, SPWMD had a stronger correlation with LVEF than initial QRS duration. In contrast to the right-sided accessory pathway groups, the left-sided accessory pathway group did not have an elevated BNP level and decreased LVEF. So, we suspect that the intraventricular LV dyssynchrony is a critical determinant in the pathogenesis of LV dysfunction. Of note, no patient had incessant tachyarrhythmia, rapid ventricular conduction of atrial fibrillation via the accessory pathway, or other arrhythmias that might have contributed to the reduced LV function at admission. Therefore, tachycardia-mediated LV dysfunction before the RFA is unlikely in our study group. Also, other factors impacting BNP levels such as age, hypertension, diabetes mellitus, renal function, and tachycardia cycle lengths did not differ among the groups.

RFA was used as a substitute for cardiac resynchronization therapy (CRT) in treating patients with type-B WPW syndrome who had intolerance for the titration of guideline oriented medical therapy. Patients with left branch bundle block often have other cardiac disorders that lead to a poor prognosis, such as mechanical dyssynchrony, mitral valve apparatus deformation, and left ventricular remodeling [20]. CRT has been established as an effective treatment for heart failure, especially in patients resistant to drug therapy [21]. Patients with type-B WPW syndrome have an accessory pathway on the right side of the heart, so their QRS complex is wide, similar to the pattern of left ventricular branch bundle block and left ventricular asynchrony. RFA has been reported to improve cardiac function in patients with right-sided accessory pathways. Tomaske et al. retrospectively confirmed the improved cardiac function of 34 symptomatic patients with type-B WPW syndrome after RFA [16]. Dai et al. also reported that RFA of accessory pathways resulted in the recovery of mechanical resynchronization and improved LV function in patients with right-sided accessory pathways [22]. However, most of these studies were in children. There have been no systematic studies evaluating this phenomenon in adult patients, only a few case reports. Iwasaku et al. performed RFA in type-B WPW syndrome patients with dilated cardiomyopathy, regardless of receiving a full dose of drug therapy, confirmed resynchronization, and improved cardiac function, mitral regurgitation, and brain natriuretic peptide levels [23].

Since the patients' mean age was 41 years, these results represent models of chronic pre-excitation syndrome. We found reversibility of LV dyssynchrony in patients with long-standing bypass tract after ablation therapy. This finding may indicate a good prognosis of LV dyssynchrony associated with pre-excitation, which is different from what has been observed in patients with heart failure. Although catheter ablation has a good success rate and safety for WPW, it is still controversial to recommend invasive catheter ablation for asymptomatic WPW patients [7]. Given the potential risk of rapid ventricular conduction of atrial fibrillation [24], severe LV dysfunction may be a further primary preventive indication for RFA in selected patients. Echocardiography should be performed in all patients with pre-excitation detected on ECG. In asymptomatic patients, decreased LV function and echocardiographic dyssynchrony may imply premature RFA procedure in patients with manifest right-sided pathways (Table 4).

The present study has several limitations. First, the sample size was small, and the data were drawn from a single-center clinical practice. Therefore, selection bias is a possibility, and the results cannot be extended to other institutions. Nevertheless, our findings could be useful for generating hypotheses and developing more extensive, multicenter studies in the future. Second, for economic reasons, we did not use tissue Doppler or cardiac magnetic resonance imaging (c-MRI) studies for assessment of LV dyssynchrony before and after RFA.

## 5. Conclusions

Anterograde conduction with a septal or right free wall accessory pathway may cause LV dyssynchrony and impair LV function, resulting in decreased LVEF and higher NT-proBNP plasma concentration. Intraventricular LV dyssynchrony appears to be responsible for the pathogenesis of LV dysfunction. Radiofrequency ablation of accessory pathways results in decreased BNP levels, normalized QRS duration, mechanical resynchronization, and improved LV function. Recovery of SPWMD was observed in all affected patients. Even in the absence of arrhythmias, decreased LV function and echocardiographic dyssynchrony may imply premature RFA procedure in patients with manifest septal or right free wall accessory pathway.

## Figures and Tables

**Table 1:** Baseline characteristics of the study population.

Parameter	Septal	Right free wall	Left free wall	Concealed	*P*
Age (years)	36 ± 16	43 ± 14	43 ± 15	43 ± 12	0.32
Female	6 (30)	7 (35)	7 (35)	9 (45)	0.795
Wt (kg)	66 ± 9	69 ± 8	69 ± 7	67 ± 10	0.566
HR (beats/min)	69 ± 10	71 ± 14	65 ± 12	74 ± 13	0.157
BUN (*μ*mol/l)	5.78 ± 1.43	5.25 ± 1.59	5.01 ± 1.69	5.24 ± 1.21	0.413
Cr (*μ*mol/l)	62.4 ± 14	70.5 ± 14.6	66.8 ± 16.7	62.4 ± 14.8	0.267
eGFR (ml/min/1.73 m^2^)	117.5 ± 17.6	107.3 ± 16.1	109.1 ± 12.5	105.2 ± 26.7	0.189
Hs-CRP (*μ*mol/l)	1.16 ± 2.22	1.01 ± 1.25	1.35 ± 2.88	0.51 ± 0.52	0.553
Cys C (*μ*mol/l)	0.79 ± 0.18	0.77 ± 0.18	0.86 ± 0.14	0.77 ± 0.16	0.265
NT-proBNP (ng/ml)	159 ± 241^#^	132 ± 129^#^	58 ± 66	48 ± 40	0.039^*∗*^

Data are given as mean ± SD or *n* (%). *P* refers to comparison between the four groups. ^*∗*^*P* < 0.05. ^#^*P* < 0.05 vs. concealed. Wt: weight; HR: heart rate; BUN: blood urea nitrogen; Cr: creatinine; eGFR: estimated glomerular filtration rate, hs-CRP: high-sensitivity C-reactive protein; Cys C: cystatin C; NT-proBNP: N-terminal pro-B-type natriuretic peptide.

**Table 2:** Electrocardiographic and echocardiographic data before and after ablation.

Parameter	Before RFCA				*P* value	After RFCA				*P* value
	Septal	Right free wall	Left free wall	Concealed		Septal	Right free wall	Left free wall	Concealed	
QRS (ms)	125 ± 19^#^	133 ± 25^#^	120 ± 14^#^	87 ± 11	<0.001^*∗*^	89 ± 10	86 ± 10	88 ± 11	87 ± 11	0.847
PR (ms)	132 ± 23^#^	116 ± 24^#^	129 ± 18^#^	145 ± 17	0.001^*∗*^	152 ± 18	146 ± 16	145 ± 12	144 ± 15	0.409
LVEDd (mm)	47 ± 5	48 ± 6	47 ± 4	45 ± 4	0.218	45 ± 3	45 ± 3	46 ± 3	44 ± 4	0.445
LVEF (%)	58 ± 4^#^	58 ± 5^#^	62 ± 1	62 ± 1	<0.001^*∗*^	63 ± 3	62 ± 2	62 ± 1	62 ± 2	0.604
IVMD (ms)	29 ± 8^#^	31 ± 5^#^	28 ± 5	26 ± 5	0.092	29 ± 5	30 ± 6	31 ± 7	26 ± 4	0.06
SPWMD (ms)	90 ± 20^#^	60 ± 16^#^	50 ± 15	43 ± 6	<0.001^*∗*^	49 ± 11	47 ± 12	46 ± 12	42 ± 5	0.242

Data are given as mean ± SD. *P* refers to comparison between the four groups. ^*∗*^*P* < 0.05. ^#^*P* < 0.05 vs. concealed. QRS: QRS duration during sinus rhythm; PR: PR interval during sinus rhythm; LVEDd: left ventricle end-diastolic dimension; LVEF: left ventricular ejection fraction; IVMD: interventricular mechanical delay; SPWMD: septal-to-posterior wall motion delay.

**Table 3:** Electrophysiological data.

Parameter	Septal	Right free wall	Left free wall	Concealed	*P* value
AH (ms)	83 ± 8	80 ± 8	78 ± 7	81 ± 9	0.201
HV (ms)	3 ± 10^*#*^	−9 ± 10^*#*^	22 ± 14^*#*^	42 ± 13	<0.001^*∗*^
TCL (ms)	364 ± 34	378 ± 39	360 ± 45	368 ± 28	0.499
ERPa (ms)	326 ± 47	336 ± 44	310 ± 49	None	0.188
ERPr (ms)	291 ± 50	292 ± 45	277 ± 46	305 ± 58	0.396

Data are given as mean ± SD. *P* refers to comparison between the four groups. ^*∗*^*P* < 0.05. ^#^*P* < 0.05 vs. concealed. AH: AH interval during sinus rhythm; HV: HV interval during sinus rhythm; TCL: tachycardia cycle length; ERPa: ERP on the anterograde accessory pathway; ERPr: ERP of the retrograde accessory pathway.

**Table 4:** Comparison of measurements in patients with septal and right free wall accessory pathways before and after ablation.

Parameter	Septal		*P* value	Right free wall		*P* value
	Before RFA	After RFA		Before RFA	After RFA	
QRS (ms)	125 ± 19	89 ± 10	<0.001^*∗*^	133 ± 25	86 ± 10	<0.001^*∗*^
PR (ms)	132 ± 23	152 ± 18	<0.001^*∗*^	116 ± 24	146 ± 16	0.001^*∗*^
LVEF (%)	58 ± 4	63 ± 3	<0.001^*∗*^	58 ± 5	62 ± 2	0.003^*∗*^
IVMD (ms)	29 ± 8	29 ± 5	0.924	31 ± 5	30 ± 6	0.881
SPWMD (ms)	90 ± 20	49 ± 11	<0.001^*∗*^	60 ± 16	47 ± 12	<0.001^*∗*^
NT-proBNP (ng/mL)	159 ± 241	78 ± 89	0.042^*∗*^	132 ± 129	52 ± 35	0.015^*∗*^

Data are given as mean ± SD. ^*∗*^*P* < 0.05. QRS: QRS duration during sinus rhythm; PR: PR interval during sinus rhythm; LVEF: left ventricular ejection fraction; IVMD: interventricular mechanical delay; SPWMD: septal-to-posterior wall motion delay; NT-proBNP: N-terminal pro-B-type natriuretic peptide.

## Data Availability

The data are available from the corresponding author upon request.
